# Molecular Pathways Regulating Macrovascular Pathology and Vascular Smooth Muscle Cells Phenotype in Type 2 Diabetes

**DOI:** 10.3390/ijms161024353

**Published:** 2015-10-14

**Authors:** Sara Casella, Alessandra Bielli, Alessandro Mauriello, Augusto Orlandi

**Affiliations:** Department of Biomedicine and Prevention, Institute of Anatomic Pathology, Tor Vergata University of Rome, Rome 00133, Italy; E-Mails: sara.casella87@icloud.com (S.C.); alessandrabielli@hotmail.it (A.B.); alessandro.mauriello@uniroma2.it (A.M.)

**Keywords:** diabetes, atherosclerosis, phenotypic changes, apoptosis, nuclear factor-κB, Akt

## Abstract

Type 2 diabetes mellitus (T2DM) is a disease reaching a pandemic proportion in developed countries and a major risk factor for almost all cardiovascular diseases and their adverse clinical manifestations. T2DM leads to several macrovascular and microvascular alterations that influence the progression of cardiovascular diseases. Vascular smooth muscle cells (VSMCs) are fundamental players in macrovascular alterations of T2DM patients. VSMCs display phenotypic and functional alterations that reflect an altered intracellular biomolecular scenario of great vessels of T2DM patients. Hyperglycemia itself and through intraparietal accumulation of advanced glycation-end products (AGEs) activate different pathways, in particular nuclear factor-κB and MAPKs, while insulin and insulin growth-factor receptors (IGFR) are implicated in the activation of Akt and extracellular-signal-regulated kinases (ERK) 1/2. Nuclear factor-κB is also responsible of increased susceptibility of VSMCs to pro-apoptotic stimuli. Down-regulation of insulin growth-factor 1 receptors (IGFR-1R) activity in diabetic vessels also influences negatively miR-133a levels, so increasing apoptotic susceptibility of VSMCs. Alterations of those bimolecular pathways and related genes associate to the prevalence of a synthetic phenotype of VSMCs induces extracellular matrix alterations of great vessels. A better knowledge of those biomolecular pathways and related genes in VSMCs will help to understand the mechanisms leading to macrovascular alterations in T2DM patients and to suggest new targeted therapies.

## 1. Introduction

Diabetes mellitus is a human metabolic disease with an economic impact on medical care. Around 10% of European population is affected by type-2 diabetes mellitus (T2DM), which develops from a complex interplay between eating habits, excess body fat, genetics, and other common disorders, such as obstructive sleep apnea. In parallel with T2DM, the incidence of insulin resistance and metabolic syndrome also increases because of the epidemic diffusion of obesity among populations of the industrialized world [1,2]. Hyperglycemia defines a full-blown T2DM. T2DM-induced pathologic changes involve diffusely the vasculature, and hyperglycemia is considered a major risk factor in promoting pathological arterial remodeling and clinically-adverse events, such as stroke and acute coronary syndrome [3]. Given those complications, it is surprising that less or minimal attention has been given to T2DM-related macrovascular disease and the role of vascular smooth muscle cells (VSMCs), the predominant cell population in the arterial wall. This review will highlight the biomolecular pathways leading to VSMC phenotypic alterations and how they contribute to pathological macrovascular remodeling in T2DM patients.

## 2. Heterogeneity of Vascular Smooth Muscle Cells and T2DM

**Figure 1:**
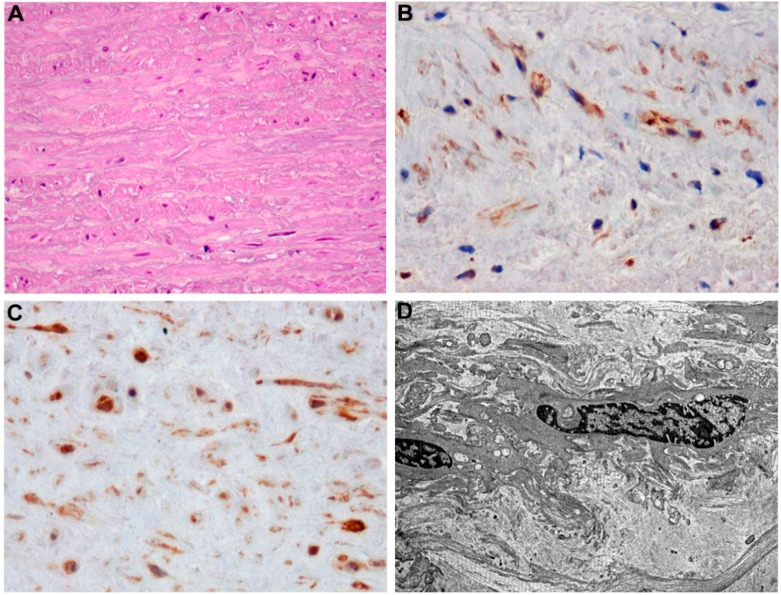
Aortopathy and diabetes: microscopic aspects. (**A**) Haematoxylin-Eosin-stained section of aortic aneurism of diabetic patient shows reduced cellularity and increased extracellular matrix accumulation; Immunohistochemical staining with 3,3-diaminobenzidine for (**B**) α-smooth actin and (**C**) smooth muscle myosin show variability of expression and shape of normal aortic VSMCs; (**D**) Ultrathin sections were counterstained with uranyl acetate and lead citrate and photographed by using a Hitachi 7100 transmission electron microscope, showing medial VSMCs with irregular nucleus surrounded from abundant extracellular matrix. Original magnification: (**A**–**C**), 400×; and (**D**), 4500×.

VSMCs in the healthy tunica media are phenotypically heterogeneous and characterized by a variable switch from a “contractile” to a dedifferentiated phenotype [4,5,6]. Contractile or differentiated phenotypes, typical of VSMCs of the normal vessels, exhibits a highly organized cytoskeleton, with defined F-actin filaments that maintain contractile function, and high levels of α-smooth muscle actin, smooth muscle myosin heavy chain and h1-calponin [7]. During the cardiovascular disorders, VSMC cytoskeletal reorganization leads to the prevalence of a synthetic phenotype. Synthetic VSMCs display alterations in organelle distribution, aberrant matrix metabolism, increased proliferation and migration [8], and expression of specific glycoproteins [9]. Key characteristics of dedifferentiation of VSMCs are nuclear enlargement, increased ribosomal content, and enlarged Golgi apparatus [10,11]. Changes of VSMC behavior are phenotypically-regulated and lead to receptor activation that regulates proliferation, migration, and survival. Functionally, VSMC dedifferentiation impairs proliferative and migratory capacity through an increased sensitivity to growth factor stimulation and mitogenic factors [10,12]. Changes in VSMC phenotype also critically influence the response to pharmacologically-active substances [13,14,15,16]. Differences in VSMC phenotype can be easily identified *in vitro*. Two cell populations can be isolated from post-injury rat aorta and carotid artery: a spindle-shaped phenotype with the classic “hill-and-valley” growth pattern, typical of cultured VSMCs, and an epithelioid phenotype growing in a monolayer with cobblestone morphology that are isolated from the neointimal tissue after balloon injury [17,18]. Epithelioid VSMCs display a different response to growth factors [18,19]. The differentiated and synthetic VSMCs phenotypes are not stable and they can switch in culture, under particular conditions, such as growth factors (*i.e.*, platelet-derived growth factors-BB (PDGF-BB), fibroblast growth factor-2 (FGF-2), transforming growth factor-β (TGF-β)) [19,20,21], or matrix molecules as collagen [22]. The metabolic milieu of insulin resistance and T2DM can progress for decades before clinical diagnosis [23]. During this period, hyperglycemia and hyperinsulinemia exert a direct effect on vascular cells, potentially causing detrimental changes in their phenotype and function (Figure 1). Increased susceptibility to cardiovascular diseases in T2DM patients suggests that a pathological phenotype of VSMCs is worthy of detailed study [24]. VSMCs from primary cultures from T2DM vessels were morphologically distinct from non-diabetic VSMCs. In particular, arterial and venous VSMCs from T2DM patients lose the typical “hill-and-valley” spindle-shaped appearance and adopt a more rhomboid phenotype [25]. VSMCs from diabetic patients show a significant increase of proliferation, adhesion and contact inhibition, associated with an increase of atheromatous process and restenosis [25]. Intimal hyperplasia appears closely linked to the synthetic phenotype of VSMCs and must be considered in order to counteract atherosclerosis and restenosis in T2DM patients [25,26].

## 3. T2DM-Induced Macrovascular Disease: Different Players

Hyperglycemia is a major risk factor for arterial diseases and leads to vascular pathology through multiple processes. High glucose concentrations activate nuclear factor-κB (NF-κB) [27,28]. The latter modulates several pro-inflammatory and pro-atherosclerotic target genes in VSMCs, endothelial cells (ECs) and macrophages. NF-κB activation is essential for early events during the progression of atherosclerotic lesions, in particular the adhesion of circulating monocytes to ECs and the transmigration in the subendothelial space. In fact, hyperglycemia-induced monocyte adhesion to cultured aortic ECs *in vitro* by NF-κB activation increased the expression of pro-inflammatory genes and endothelial adhesion molecules, in particular vascular cell adhesion molecule 1 (VCAM-1) and monocyte chemoattractant protein-1 (MCP-1) [29,30]. MCP-1 is an important chemotactic factor for monocytes, mainly secreted by ECs, macrophages and adipocytes [31]. MCP-1 and its receptor CC chemokine receptors2 (CCR2) are important players in the monocyte recruitment. MCP-1 and CCR2 are likewise important for the onset of cardiovascular pathology and the negative effect of these circulating factors induces EC dysfunction, adhesion of monocytes and subsequent vascular remodeling with foam cells accumulation and plaque development. Expression of adhesion molecules and MCP-1 characterizes the inflammatory response of ECs *in vitro* [32,33] and appears up-regulated in aged great vessels [34]. After migration into the subendothelial space, monocytes differentiate into macrophages that are increased in the artery wall of diabetic patients [35]. Experimental data indicated that in response to hyperglycemia, macrophages can also locally proliferate [36]. Increased NF-κB activity also characterizes VSMC exhibiting a synthetic phenotype [16] and NF-κB inhibition promotes VSMC apoptosis [37]. NF-κB also regulates the pro-apoptotic response of intimal VSMCs to neuromediators, as pro-nerve growth factor [38]. Mitochondrial nicotinamide adenine dinucleotide phosphate oxidase (NADPH) activity, in particular the Nox4 isoform, is a dominant mechanism for endothelial reactive oxygen species (ROS) production [32,39]. Endothelial cells under high levels of glucose showed an increase expression of Nox2 and Nox4 isoform, and greater Rac-1 activity; the latter is a small G protein, important for intracellular NADPH oxidase assembly and activation [40]. T2DM induced Rac-1 activity through its translocation from cytoplasm to plasma membrane, promoting NADPH oxidase subunit assembly [41]. The latter is a necessary mechanism to trigger the ROS-generating NADPH oxidase enzymatic system. In diabetic mice vessels it was found an increased Rac-1 and NADPH oxidase activity, demonstrating that molecular processes leading to NADPH oxidase-dependent generation of ROS in T2DM implicate Rac-1 activation [41]. So, oxidative stress-induced vascular endothelial dysfunction and Rac-1 activity depend on hyperglycemic conditions of diabetic patients [41]. The selective inhibition of Rac-1, blocking a key step in oxidative stress generation, could reduce hyperglycemia-induced vascular damage and consequently the cardiovascular risk in T2DM [41]. Together, hyperglycemia and ROS accumulation inhibit the endothelial production of nitric oxide, leading to endothelial-dependent relaxation dysfunction that characterizes vascular pathology [42,43]. In diabetic patients, ROS accumulation contributes to LDL modification and oxidation [44]. Epidemiologic data indicate that in human subjects, LDL are the most atherogenic lipoproteins, particularly susceptible to oxidative modifications from other atherogenic risk, such as cigarette smoke, diabetes, and insulin resistance [45]. In diabetic patients, insulin resistance also promotes the progression of plaque to vulnerable lesions [46]. Lipid accumulation further stimulates recruitment of macrophages and other inflammatory cells [47], contributing to a state of vascular inflammation. The latter induces intimal VSMC accumulation, which display macrophagic activity and increased synthetic activity with extracellular collagen deposition [48] in a sort of accelerated vascular aging [49,50]. Diabetes-induced vascular alterations share same aspects with age-related macroangiopathy, and both represent a risk factor for atherosclerosis [49,51]. Aging potentiates other atherogenic risk factors, including diabetes [52] and VSMCs, also drive the age-related vascular changes [53]. The age-related diffuse intimal thickening in great vessels the principal site for the progression of atherosclerotic lesions [49,51]. VSMCs from old donors show increased expression of stem markers [54]. Vascular expression of flt-1+ and c-kit+ influences VSMC properties [55]. In particular, flt-1 signaling regulates NF-κB-mediated cell survival [56], in line with the hypothesis that VSMCs with a stem phenotype promote arterial remodeling [54,57]. Since it has been hypothesized that circulatory precursors can be beneficial in the stabilization of plaques [58]. It is suggestive to hypothesize that circulating and resident cells with stem phenotype play a different role in aortic remodeling observed in T2DM patients [59].

## 4. VSMCs and T2DM-Related Atherogenic Risk Factors

Similar to that observed during the development of atherosclerotic lesions, VSMCs undergo a variety of T2DM changes and display a different phenotype in great vessels. A better knowledge of factors regulating VSMC phenotype is crucial to suggest new targeted therapies to prevent aortopathy in T2DM patients.

### 4.1. Hyperglycemia

High glucose concentrations induce VSMC proliferation and play a pivotal role in the progression of diabetic vascular alterations [60]. It appears relevant to investigate the molecular mediators in response to increased blood high glucose levels as a potential therapeutic target. Interferon regulatory factor-1 (IRF-1) is a molecular mediator of vascular diseases. Several studies provided evidences that IRF-1 reduces vascular cell growth under normal glucose conditions, whereas at high concentrations IRF-1 promotes VSMC proliferation [61]. Other studies indicated that ROS accumulation influences proliferation by activating cyclin/CDK [62]. ROS accumulation also induces endothelial dysfunction [32,39]. High glucose-induced stimulation increases intracellular ROS and stimulates the activation of ERK 1/2, a mitogen-activated protein kinase essential for VSMC growth [63]. IRF-1 over-expression promotes ERK-1 activation under high-glucose condition. It is likely that the IRF-1-mediated pathway sustains hyperglycemia-dependent VSMC proliferation [63]. Elevated glucose concentration also stimulate extracellular matrix synthesis and accumulation [64], likely mediated by the activity of TGF-β [65] and its downstream mediator the connective tissue growth factor, that regulates vascular fibrosis [65]. High glucose levels increased connective tissue growth factor protein and mRNA in VSMCs and its deletion by siRNA inhibited the high glucose-induced VSMC proliferation [66]. The involvement of connective tissue growth factor in high glucose-induced proliferation suggests that its down-regulation may prevent abnormal VSMC growth in the vessels of diabetic patients.

### 4.2. Advanced Glycation End-Product

Several hypotheses aim to explain the increase of VSMC proliferation in pathological vessels. Proteins and lipids exposed to high glucose levels generate advanced glycation end-products (AGEs) as protein cross-links, flurophors, and low molecular-weight residues [42]. AGE accumulation induces the increased production of ROS. ROS accumulation plays a central role in vascular disease, including atherosclerosis and restenosis. AGE accumulation activated NF-κB in many cell types, in particular in VSMCs [67]. Moreover, AGEs also induce mitogen activated protein kinase (MAPKs) activation [68]. The role of AGE receptors (RAGEs) has been also investigated. RAGEs and galectin-3 have been linked to the progression of atherosclerosis [69,70]. VSMCs proliferation is likely mediated via galectin-3 and abnormal AGE-galectin-3 interaction has been linked to macroangiopathy in T2DM patients [71].

### 4.3. Hyperinsulinemia and Insulin Growth Factor

Hyperinsulinemia is an important factor for the plaque formation in T2DM patients. Insulin displayed mitogenic effects on human aortic VSMCs [72] and insulin growth factor-1 (IGF-1) activated insulin-dependent proliferation of human VSMC [73] through various pathways, including MAPK signaling [74], that in turn increases human VSMC chemotaxis [75]. IGF-1 has been also documented to influence VSMC survival [76]. IGF-1 binds to its receptor IGF-1R with high affinity, leading to the activation of IGF-1R tyrosine kinase, which in turn induces pro-survival and growth signals. Insulin resistance of VSMCs in T2DM patients is linked to angiotensin II-mediated vasculopathy [77]. In the vascular system, NO activity is able to antagonize angiotensin II. The latter is able to stimulate ROS production [77].However, the relationship between angiotensin II and insulin resistance has not been established. A prolonged oxidative stress and increased angiotensin II may cause insulin resistance thorough ROS-mediated activation of insulin receptor substrate-1 (IRS-1) of VSMCs. IRS-1 phosphorylation reduced phosphatidylinositol 3-kinase activity (PI3K) and abolished insulin-induced activation of Akt [77]. This effect induces the decrease of GLUT4 (glucose transporter type 4) translocation to the plasma membrane, which causes a reduced glucose uptake in VSMCs, demonstrating that insulin-mediated glucose uptake in VSMCs is linked to IRS-1/PI3K/Akt pathway, similarly to other insulin-sensitive tissues [78]. In addition, increased ROS and angiotensin II production are responsible for the increased VSMC proliferation, vascular inflammation and extracellular matrix deposition [79]. Persistent exposure to IGF-1 stimulates growth and migration of VSMCs. In advanced plaques, intimal VSMC hyperplasia may reduce the plaque vulnerability to rupture and it helps to stabilize atherosclerotic lesions [80].Biological effects of insulin on VSMCs are mediated by IGF-1R [81]. IGF-1R expression was down-regulated in aortas of diabetic mice, likely as the consequence an alteration in insulin responsiveness [82]. When IGF-1R is down-regulated, insulin induces Akt and ERK 1/2 phosphorylation as well as glucose uptake [82]. Akt and ERK 1/2 play a relevant regulatory role on survival and vasculogenic properties of human adipose tissue-derived stem cells [33,83]. Adventitial cells may be the source of stem cells with vasculogenic properties [17]. Enhanced insulin signaling in VSMCs results in the suppression of TNF-α-induced NF-κB activation and MCP-1 expression [82]. These results suggest that manipulation of IGF-1R activity is a therapeutic target to improve insulin sensitivity and also indicate that insulin plays a potentially anti-inflammatory role in the vasculature. 

## 5. MicroRNAs and Diabetic Macrovascular Pathology

MicroRNAs (miRs) represent a class of new-coding small RNA species that promote mRNA cleavage and/or translational repression through base-paring to the 3ʹ untranslated region (UTR) of target mRNAs [84]. Recently miR-133a has been reported to be expressed in VSMCs and to regulate IGF-1R expression [58]. In the aorta, miR-133a was reduced in ApoE^−/−^ mice [58]. It is expected that VSMCs may be susceptible to apoptosis when the expression of the inhibitor of apoptosis IGF-1R, is compromised, likely the consequence of low miR-133a level in VSMCs of atherosclerotic aortas [58]. MiR-133a/IGF-1R promotion of VSMC proliferation and differentiation may help to prevent plaque rupture and adverse clinical events [58]. IGF-1 increases the expression of ribonucleoprotein domain family member 6 (LARP6) in VSMCs, resulting in an increased binding of LARP6 to COL1a1 and COL1a2 mRNAs, with enhanced synthesis of collagen type I and extracellular matrix accumulation of mature collagen by promoting fibril maturation, with a more stable plaque phenotype [85]. These data indicate that the regulatory mechanisms of IGF-1 during the development of atherosclerotic plaques are quite different from those of macrovascular pathology in T2DM patients. Hyperglycemia can work in synergy with hyperinsulinemia and other factors to promote atherosclerotic plaque progression in diabetic patients. Growth-promoting effects of high glucose level on VSMCs have been described in human and rat aorta and human umbilical artery cultures [24,86,87]. These results were not confirmed in experiments using porcine SMCs, where high glucose levels did not stimulate proliferation neither via synergism with other SMC mitogens, such as PDGF [24,88].

## 6. Adipose Tissue and Diabetes-Related Macrovascular Dysfunction

Adipose tissue has a central role in the progression of a systemic low-grade inflammatory condition that favors vascular dysfunction in obese subjects. The local production of adipokines and chemokines by fat cells suggests a novel interplay between obesity and vascular pathology [89]. In addition, chemotactic adipokines modulate the immune cell infiltration in the adipose tissue stimulating atherosclerotic process [90]. Accumulating evidence showed that adipose tissue cells secrete adipokines and favor the development of cardiovascular complications in T2DM patients, an increase of superoxide affects cardiomyocytes and VSMC function [91]. However, adiponectin plasma level was significantly lower in adult subjects with obesity [92] and T2DM [93]. Adipose tissue-secreted adipokines promote vascular dysfunction associated with obesity and vascular pathology. Interestingly, vascular endothelial growth factor (VEGF) released from visceral adipose tissue, seemed linked to increased VSMC proliferation [94]. VEGF activity is mediated by specific receptors, VEGFR-1 and VEGFR-2 [95,96]. Increased expression of VEGFR-1 is associated to phenotype changes of VSMCs and influences biological properties and pharmacological response [54,56,97,98]. Adiponectin also induces NO production by increasing of endothelial nitric oxide synthase (eNOS) phosphorylation [99]; eNOS is critically involved in microvascular dysfunction, since it induces overproduction and release of O_2_^−^ [100]. Diabetes or obesity are also associated to increased levels of free fatty acids, that increase intracellular diacylglycerol concentration in several tissues, including renal glomeruli from diabetic animals and VSMCs. Intracellular diacylglycerol promotes membrane-associated protein kinase C activity that leads to increased expression of cyclooxygenase and vasoconstrictor thromboxane A2, whereas vasodilator prostacyclin production decreases [101]. Overall, those mechanisms accelerate vascular dysfunction in people with obesity-associated insulin resistance and in diabetes.

## 7. Conclusions

**Figure 2:**
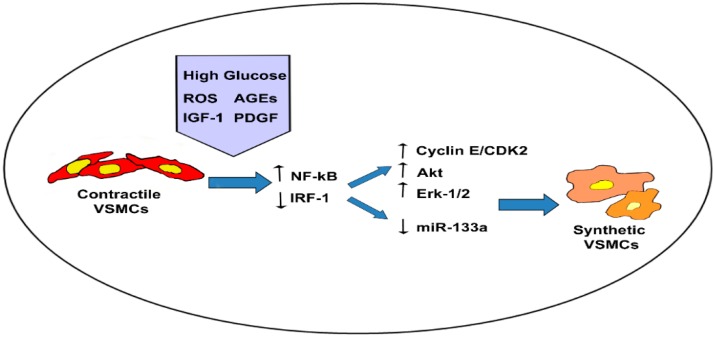
Phenotypic switch of VSMCs in diabetic aortopathy. Schematic representation of diabetes-related intracellular signaling involved in the switch from contractile to synthetic phenotype of vascular smooth muscle cells (VSMCs) in great vessels.

During the last years, many studies have been focused on the investigation of molecular mechanisms regulating VSMC phenotype in pathological vessels. Hyperglycemia and hyperinsulinemia induce changes of VSMC phenotype that is critical for the progression of T2DM macrovascular pathology. Investigation of alterations of VSMCs phenotype in T2DM patients allowed the recognition of specific intracellular pathways supporting pathological macrovascular remodeling, in particular NF-κB, Akt and ERK-related genes, as summarized in Figure 2. Moreover, miR-RNAs represent a promising field of research to discover new mechanism of VSMC homeostasis in T2DM pathological vessels. The next step will be to discover therapeutic agents capable to correct the activation of T2DM-related biomolecular pathways in VSMCs in order to prevent macrovascular pathology.
